# Supplementary material for: Patterns of Dietary Fatty Acids and Fat Spreads in Relation to Blood Pressure, Lipids and Insulin Resistance in Young Adults: A Repeat Cross-Sectional Study
Source: Nutrients. 2025 Feb 28;17(5):869. doi: 10.3390/nu17050869 (PMC11901904; doi:10.3390/nu17050869)

Figure S1: The CONSORT flow diagram.

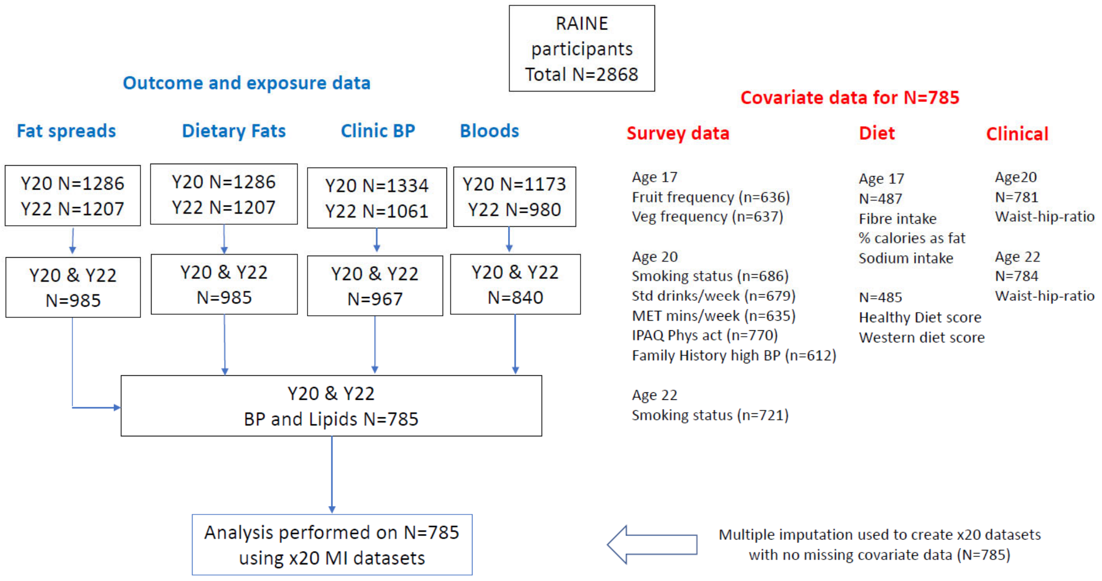

Figure S2: Dietary fatty acid clusters with cluster number and size.

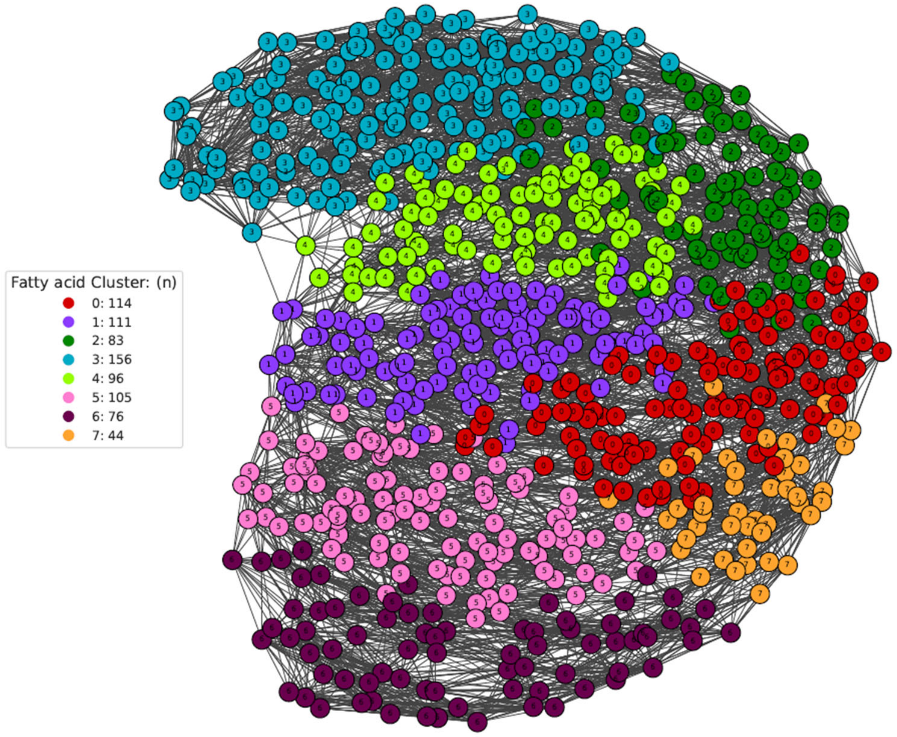

Figure S3: Dietary fat spread clusters with cluster number and size.

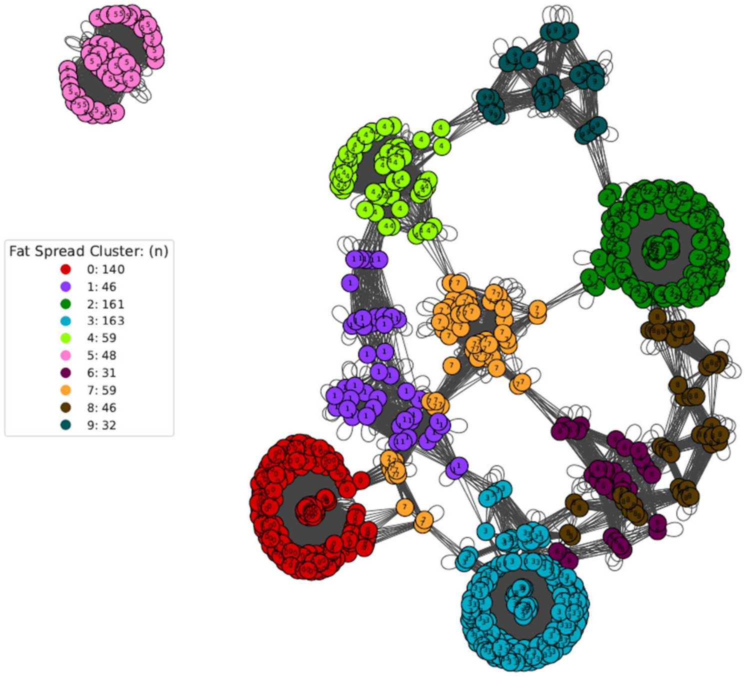

Figure S4: Percentage use of spreads by fatty acid cluster

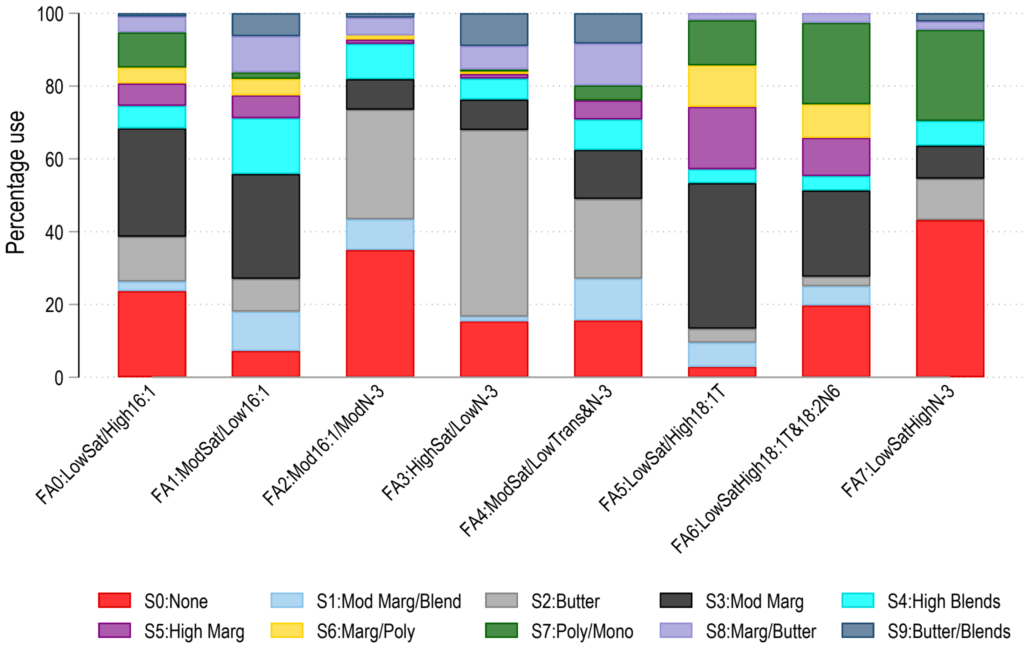

Figure S5: Coefficient plot showing mean difference (95% CI) in clinic pulse pressure for each cluster versus reference category clusters (n=786). Coefficients were estimated from regression models for males and females separately.

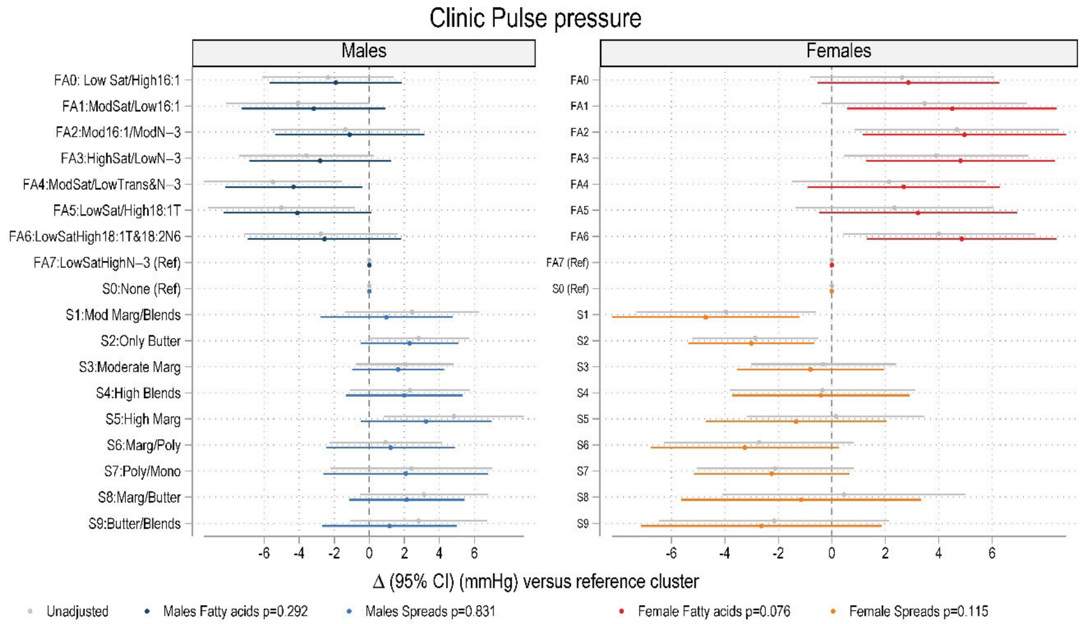

Adjusted model includes age, sex, WHR, fruit intake, vegetable intake, fibre, sodium, Healthy diet score, Western diet score, familyHx hypertension, alcohol, METS/week, physical activity category and smoking. P-values are for the adjusted model.

Figure S6: Coefficient plot showing mean difference (95% CI) in HOMA for each cluster versus reference category clusters (n=786). Coefficients were estimated from regression models for males and females separately.

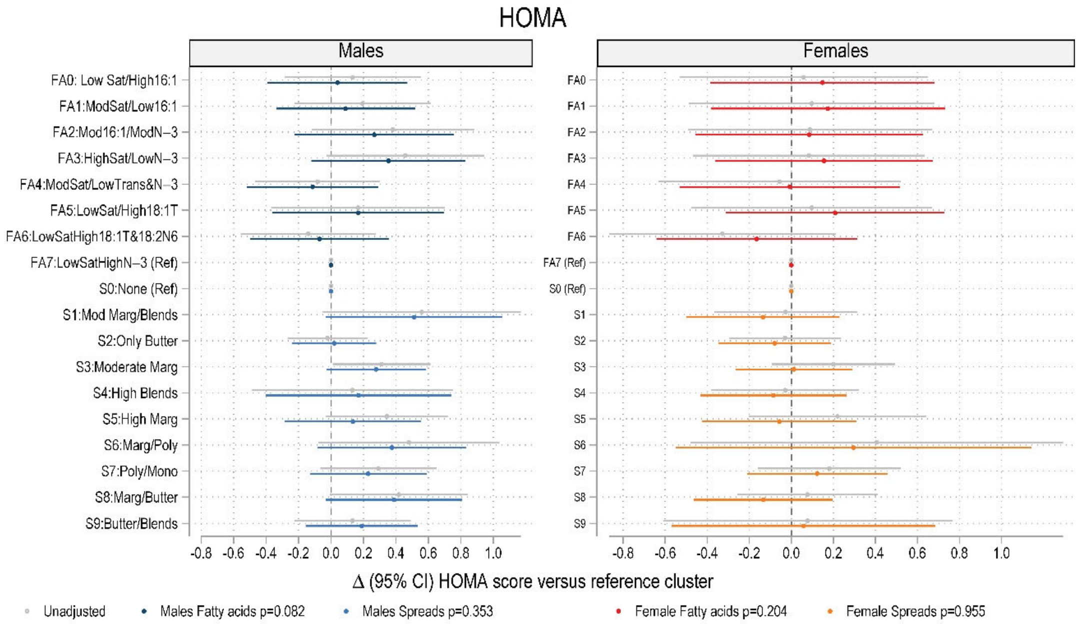

Adjusted model includes age, sex, WHR, fruit intake, vegetable intake, fibre, sodium, Healthy diet score, Western diet score, familyHx hypertension, alcohol, METS/week, physical activity category and smoking. P-values are for the adjusted model.

Figure S7: Coefficient plot showing mean difference (95% CI) in total cholesterol concentrations for each cluster versus reference category clusters (n=786). Coefficients were estimated from regression models for males and females separately.

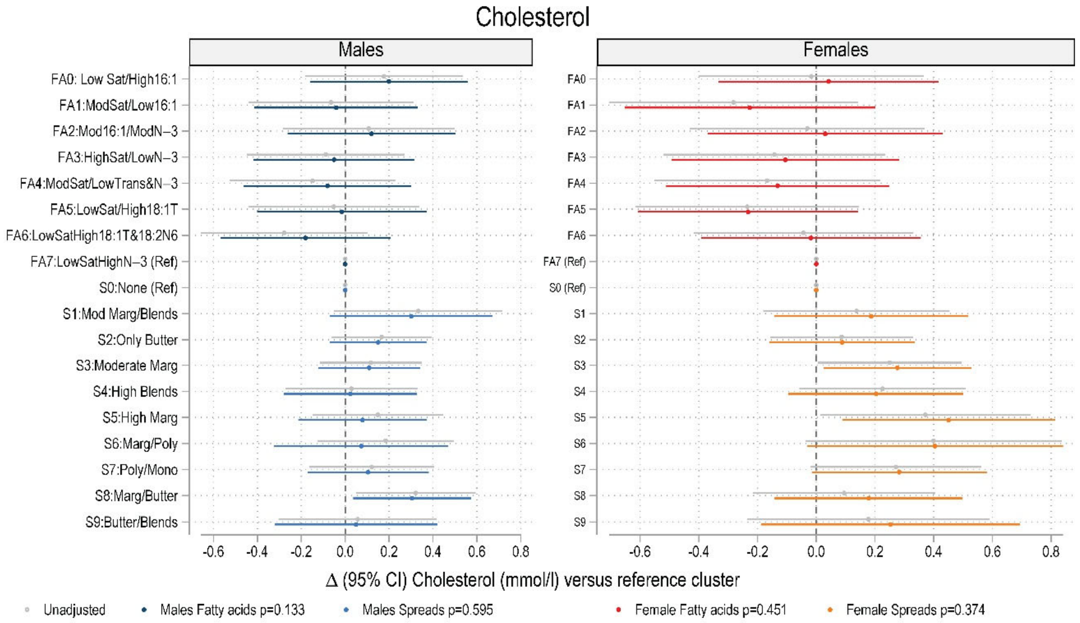

Adjusted model includes age, sex, WHR, fruit intake, vegetable intake, fibre, sodium, Healthy diet score, Western diet score, familyHx hypertension, alcohol, METS/week, physical activity category and smoking. P-values are for the adjusted model.

Figure S8: Coefficient plot showing ratio change (95% CI) in HDL cholesterol concentration geometric means for each cluster versus reference category clusters (n=786). Coefficients were estimated from regression models for males and females separately.

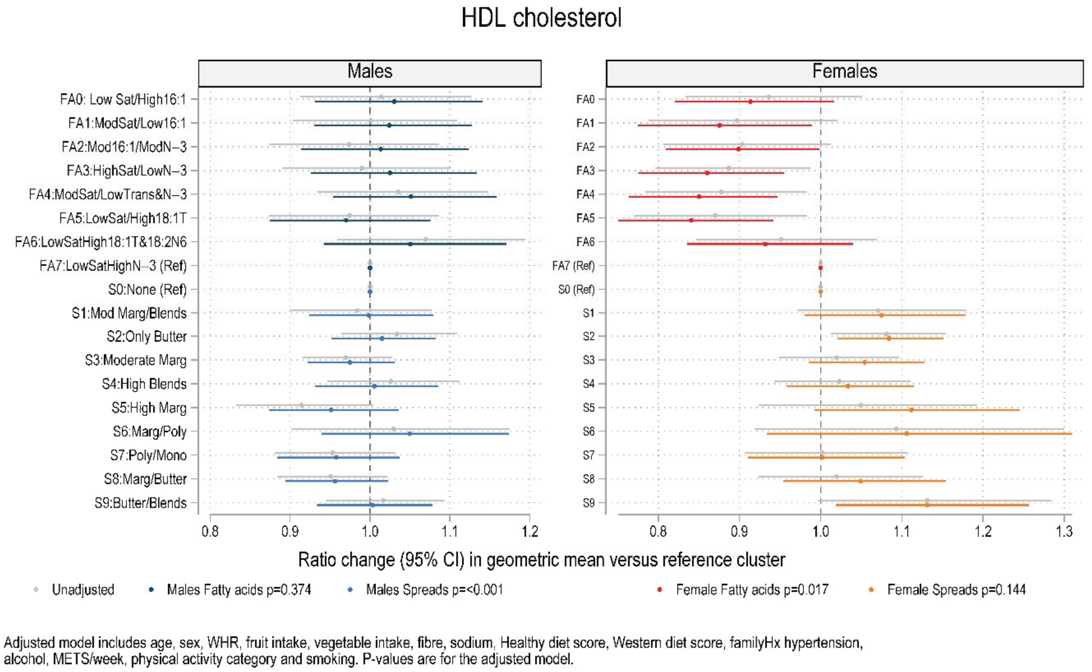

Figure S9: Coefficient plot showing ratio change (95% CI) in Non-HDL cholesterol concentration geometric means for each cluster versus reference category clusters (n=786). Coefficients were estimated from regression models for males and females separately.

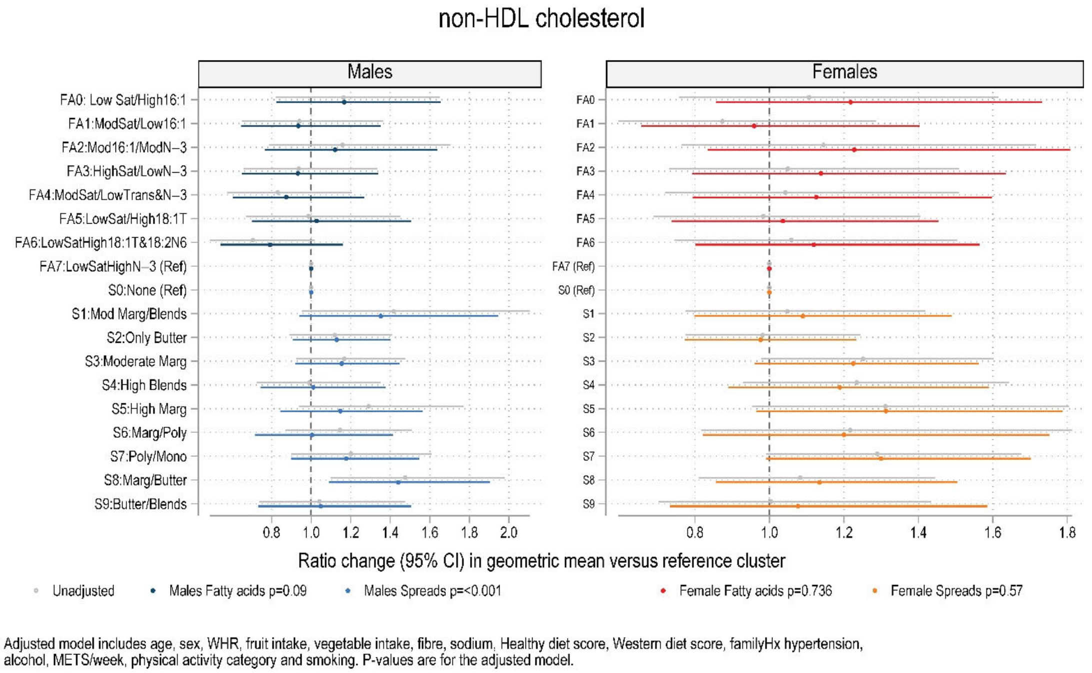

Supplement: Supplementary file 1 [file nutrients-17-00869-s001.zip › nutrients-3487112-supplementary/nutrients-3487112-supplementary figures.pdf]
