# Supplementary material for: Patterns of Dietary Fatty Acids and Fat Spreads in Relation to Blood Pressure, Lipids and Insulin Resistance in Young Adults: A Repeat Cross-Sectional Study
Source: Nutrients. 2025 Feb 28;17(5):869. doi: 10.3390/nu17050869 (PMC11901904; doi:10.3390/nu17050869)
Supplement: Supplementary file 1 [file nutrients-17-00869-s001.zip › nutrients-3487112-supplementary/Supplementary File S1.pdf]

Generation of the fatty acid and fat spread clusters

K-NN matrix construction

A K-NN matrix with cosine similarity was constructed using the scikit-learn package for machine learning in Python [1] using a 2-step procedure to create a matrix of subject-to-subject similarities. First, we applied L2 normalization. For example, for the fatty acid data, the n=31 fatty acids x_j for each subject were mean-centred and standardized such that together the new fatty acid features had zero mean and unit length.

L2 normalisation: ($\sum_{j=1}^{31}[x_j\ new]^2$) / √31= 1.00

Next, the K-NN algorithm was applied to determine subject similarity based on the 31 fatty acids. We used the BallTree method from the sklearn.neighbour's library in Python and trialled different values of K including, K=10, 20, 30 and 40. We chose k=20 nearest neighbours since this value maximised the separation of clusters in the community detection step.

Network creation

Next, the Euclidean distances between each pair of nodes (subjects) from the K-NN were transformed to cosine similarities for use as edge weights in the creation of the subject-subject network. The cosine similarity between each subject pair A and B is calculated from the Euclidean distances and is defined as:

Cosine similarity (A, B) ≡ $1 - \frac{\theta(A,B)}{\pi}$

where θ(A, B) is the angle between the vectors for subjects A and B. Since A and B are also of unit length (L2 normalized), this angle is related to the Euclidean distance D_{euc}(A, B) according to:

$\theta(A, B) = \mathrm{Cos}^{-1}\left[1 - \frac{D_{euc}(A,B)^2}{\pi}\right]$

or: $D_{euc}(A, B) = \sqrt{2 - 2\,Cos\theta(A, B)}$

Then,

Network edge-weights = $1 - \theta(A, B) = 1 - \mathrm{Cos}^{-1}\left[1 - \frac{D_{euc}(A,B)^2}{\pi}\right]$

A subject-to-subject similarity graph for the n=785 subjects was then plotted using the 785 x 20 edge-weight matrix containing the weighted edges for the 20 nearest connections of each subject. The weights of the connections were the previously estimated Euclidean distances with cosine transformation. Network layouts were created using the force-directed (Fruchterman–Reingold) layout algorithm [2] as implemented in the igraph Python package [3].

Louvain Community detection

The Louvain algorithm [4] was used for detection of communities within the K-NN network. The Louvain method partitions the nodes of the network into communities c1, c2, c3…., such that the network modularity score Is maximised. The score is a measure that quantifies edge density within communities compared to edge sparseness between communities.

Network modularity score Q = $\frac{1}{2m}\left[\sum_{i,j} A_{i,j} - \frac{k_i \cdot k_j}{2m}\right]\delta(c_i, c_j)$

In the above formula, $A_{i,j}$ is the edge weight between nodes i and j, k_i is the degree of node i (that is, the sum of the weights of all the links emanating from node i), m is the overall sum of the weights, m = $\frac{1}{2}\sum_{i,j} A_{i,j}$ and $\delta(c_i, c_j)$ is the Kronecker delta function. The network modularity score is the difference between the number of links connecting nodes within the same community and the expected number of links within a network based on randomly shuffled links.

1. Pedregosa, F.; Varoquaux, G.; Gramfort, A.; Michel, V.; Thirion, B.; Grisel, O.; Blondel, M.; Prettenhofer, P.; Weiss, R.; Dubourg, V., et al. Scikit-learn: Machine Learning in Python. *J. Mach. Learn. Res.* **2011**, *12*, 2825–2830.

2. Fruchterman, T.M.J.; Reingold, E.M. Graph drawing by force-directed placement. *Software: Practice and Experience* **1991**, *21*, 1129-1164, doi:https://doi.org/10.1002/spe.4380211102.
3. Csárdi, G.; Nepusz, T. The igraph software package for complex network research.
4. Blondel, V.D.; Guillaume, J.-L.; Lambiotte, R.; Lefebvre, E. Fast unfolding of communities in large networks. *Journal of Statistical Mechanics: Theory and Experiment* **2008**, *2008*, P10008, doi:10.1088/1742-5468/2008/10/P10008.
