# Supplementary material for: Patterns of Dietary Fatty Acids and Fat Spreads in Relation to Blood Pressure, Lipids and Insulin Resistance in Young Adults: A Repeat Cross-Sectional Study
Source: Nutrients. 2025 Feb 28;17(5):869. doi: 10.3390/nu17050869 (PMC11901904; doi:10.3390/nu17050869)
Supplement: Supplementary file 1 [file nutrients-17-00869-s001.zip › nutrients-3487112-supplementary/Supplementary File S2. STROBE_checklist_cross-sectional.pdf]

STROBE Statement—Checklist of items that should be included in reports of ***cross-sectional studies***

	Item No	Recommendation	Page No
**Title and abstract**	1	(*a*) Indicate the study's design with a commonly used term in the title or the abstract	Lines 24-44
		(*b*) Provide in the abstract an informative and balanced summary of what was done and what was found	Lines 26-44.
**Introduction**			
Background/rationale	2	Explain the scientific background and rationale for the investigation being reported	Lines 48-82
Objectives	3	State specific objectives, including any prespecified hypotheses	Lines 82-85
**Methods**			
Study design	4	Present key elements of study design early in the paper	Lines 95-97.
Setting	5	Describe the setting, locations, and relevant dates, including periods of recruitment, exposure, follow-up, and data collection	Lines 87-100.
Participants	6	(*a*) Give the eligibility criteria, and the sources and methods of selection of participants	Lines 92-97.
Variables	7	Clearly define all outcomes, exposures, predictors, potential confounders, and effect modifiers. Give diagnostic criteria, if applicable	Outcomes: Lines 117-129. Exposures. Lines 130-151 and 173-174.
Data sources/ measurement	8*	For each variable of interest, give sources of data and details of methods of assessment (measurement). Describe comparability of assessment methods if there is more than one group	Lines 102-115.<br>Lines 117-151.
Bias	9	Describe any efforts to address potential sources of bias	Page 3 section 2.1 (confounding) and Page 5 section 2.6 (multiple imputation).
Study size	10	Explain how the study size was arrived at	Lines 95-100.
Quantitative variables	11	Explain how quantitative variables were handled in the analyses. If applicable, describe which groupings were chosen and why	Lines 163-170.
Statistical methods	12	(*a*) Describe all statistical methods, including those used to control for confounding	Lines 171-183.
		(*b*) Describe any methods used to examine subgroups and interactions	Lines 172-173.
		(*c*) Explain how missing data were addressed	Lines 183-190.
		(*d*) If applicable, describe analytical methods taking account of sampling strategy	Not applicable.
		(*e*) Describe any sensitivity analyses	Not applicable.
**Results**			
Participants	13*	(a) Report numbers of individuals at each stage of study—eg numbers potentially eligible, examined for	Supplementary Figure 1

		eligibility, confirmed eligible, included in the study, completing follow-up, and analysed	
		(b) Give reasons for non-participation at each stage	Supplementary Figure 1
		(c) Consider use of a flow diagram	Supplementary Figure 1
Descriptive data	14*	(a) Give characteristics of study participants (eg demographic, clinical, social) and information on exposures and potential confounders	Lines 194-220.
		(b) Indicate number of participants with missing data for each variable of interest	Supplementary Figure 1.
Outcome data	15*	Report numbers of outcome events or summary measures	Lines 194-196.
Main results	16	(*a*) Give unadjusted estimates and, if applicable, confounder-adjusted estimates and their precision (eg, 95% confidence interval). Make clear which confounders were adjusted for and why they were included	Figures 3, 4, 5 and supplementary figures S5-S9.<br>Lines 175-177.
		(*b*) Report category boundaries when continuous variables were categorized	Lines 223-229, 238-242 and Figures S2 and S3.
		(*c*) If relevant, consider translating estimates of relative risk into absolute risk for a meaningful time period	Not applicable.
Other analyses	17	Report other analyses done—eg analyses of subgroups and interactions, and sensitivity analyses	Not applicable.
**Discussion**			
Key results	18	Summarise key results with reference to study objectives	Lines 310-321.
Limitations	19	Discuss limitations of the study, taking into account sources of potential bias or imprecision. Discuss both direction and magnitude of any potential bias	Lines 375-397.
Interpretation	20	Give a cautious overall interpretation of results considering objectives, limitations, multiplicity of analyses, results from similar studies, and other relevant evidence	Lines 400-408.
Generalisability	21	Discuss the generalisability (external validity) of the study results	Lines 381-383.
**Other information**			
Funding	22	Give the source of funding and the role of the funders for the present study and, if applicable, for the original study on which the present article is based	Lines 430-446.

*Give information separately for exposed and unexposed groups.

Note: An Explanation and Elaboration article discusses each checklist item and gives methodological background and published examples of transparent reporting. The STROBE checklist is best used in conjunction with this article (freely available on the Web sites of PLoS Medicine at http://www.plosmedicine.org/, Annals of Internal Medicine at http://www.annals.org/, and Epidemiology at http://www.epidem.com/). Information on the STROBE Initiative is available at www.strobe-statement.org.
